# Molecular Pathway and Immune Profile Analysis of IPMN-Derived Versus PanIN-Derived Pancreatic Ductal Adenocarcinomas

**DOI:** 10.3390/ijms252313164

**Published:** 2024-12-07

**Authors:** Margaret A. Park, Kristyn Gumpper-Fedus, Somashekar G. Krishna, Maria C. Genilo-Delgado, Stephen Brantley, Phil A. Hart, Mary E. Dillhoff, Maria F. Gomez, Toni L. Basinski, Shaffer R. Mok, Anjuli K. Luthra, Jason B. Fleming, Amir Mohammadi, Barbara A. Centeno, Kun Jiang, Aleksandra Karolak, Daniel Jeong, Dung-Tsa Chen, Paul A. Stewart, Jamie K. Teer, Zobeida Cruz-Monserrate, Jennifer B. Permuth

**Affiliations:** 1 Department of Gastrointestinal (GI) Oncology, Moffitt Cancer Center, Tampa, FL 33612, USA; margaret.park@moffitt.org (M.A.P.); maria.genilodelgado@moffitt.org (M.C.G.-D.); maria.gomez@moffitt.org (M.F.G.); toni.basinski@moffitt.org (T.L.B.); shaffer.mok@moffitt.org (S.R.M.); anjuli.luthra@moffitt.org (A.K.L.); amir.mohammadi@moffitt.org (A.M.); 2 Department of Biostatistics and Bioinformatics, Moffitt Cancer Center, Tampa, FL 33612, USA; dung-tsa.chen@moffitt.org (D.-T.C.); paul.stewart@moffitt.org (P.A.S.); jamie.teer@moffitt.org (J.K.T.); 3 Division of Gastroenterology, Hepatology and Nutrition, The Ohio State University Comprehensive Cancer Center, The Ohio State University Wexner Medical Center, Columbus, OH 43210, USA; kristyn.gumpper-fedus@osumc.edu (K.G.-F.); somashekar.krishna@osumc.edu (S.G.K.); philip.hart@osumc.edu (P.A.H.); 4 Department of Pathology, Moffitt Cancer Center, Tampa, FL 33612, USA; stephen.brantley@moffitt.org (S.B.); barbara.centeno@moffitt.org (B.A.C.); kun.jiang@moffitt.org (K.J.); 5 Department of Surgery, Division of Surgical Oncology, The Ohio State University Comprehensive Cancer Center, Columbus, OH 43210, USA; mary.dillhoff@osumc.edu; 6 Department of Cancer Epidemiology, Moffitt Cancer Center, Tampa, FL 33612, USA; 7 Department of Surgery, UT Southwestern Medical Center, Dallas, TX 75390, USA; jason.fleming@utsouthwestern.edu; 8 Department of Machine Learning, Moffitt Cancer Center, Tampa, FL 33612, USA; aleks.karolak@moffitt.org; 9 Department of Radiology, Moffitt Cancer Center, Tampa, FL 33612, USA; daniel.jeong@moffitt.org

**Keywords:** pancreatic ductal adenocarcinoma, IPMN, PanIN, transcriptomics

## Abstract

Intraductal papillary mucinous neoplasms (IPMN) are commonly detected pancreatic cysts that may transform into pancreatic ductal adenocarcinoma (PDAC). Predicting which IPMNs will progress to PDAC remains a clinical challenge. Moreover, identifying those clinically evident IPMNs for which a surveillance approach is best is a dire clinical need. Therefore, we aimed to identify molecular signatures that distinguished between PDAC with and without clinical evidence of an IPMN to identify novel molecular pathways related to IPMN-derived PDAC that could help guide biomarker development. Data from the Oncology Research Information Exchange Network (ORIEN) multi-institute sequencing project were utilized to analyze 66 PDAC cases from Moffitt Cancer Center and The Ohio State University Wexner Medical Center, for which tumor whole transcriptome sequencing datasets were generated. Cases were classified based on whether a tumor had originated from an IPMN (*n* = 16) or presumably through the pancreatic intraepithelial neoplasia (PanIN) pathway (*n* = 50). We then performed differential expression and pathway analysis using Gene-Set Enrichment Analysis (GSEA) and Pathway Analysis with Down-weighted Genes (PADOG) algorithms. We also analyzed immune profiles using the Tumor-Immune Microenvironment Deconvolution web portal for Bulk Transcriptomics (TIMEx). Both GSEA and TIMEx indicate that PanIN-derived PDAC tumors enrich inflammatory pathways (complement, hedgehog signaling, coagulation, inflammatory response, apical surface, IL-2/STAT5, IL-6/STAT3, EMT, KRAS signaling, apical junction, IFN-gamma, allograft rejection) and are comparatively richer in almost all immune cell types than those from IPMN-derived PDAC. IPMN-derived tumors were enriched for metabolic and energy-generating pathways (oxidative phosphorylation, unfolded protein response, pancreas beta cells, adipogenesis, fatty acid metabolism, protein secretion), and the most significantly upregulated genes (padj < 0.001) included mucin 2 (MUC2) and gastrokine-2 (GKN2). Further, the metabolic-linked gene signature enriched in the IPMN-derived samples is associated with a cluster of early-stage and long-survival (top 4th quartile) PDAC cases from The Cancer Genome Atlas (TCGA) expression database. Our data suggest that IPMN-derived and PanIN-derived PDACs differ in the expression of immune profiles and metabolic pathways. These initial findings warrant validation and follow-up to develop biomarker-based strategies for early PDAC detection and treatment.

## 1. Introduction

Although great strides have been made in improving survival for many cancer types, the prognosis for pancreatic ductal adenocarcinoma (PDAC) remains grim, with a 5-year relative survival rate of only 13% [1,2,3,4]. Moreover, both incidence and mortality for PDAC are rising, and this disease is projected to become the second leading cause of cancer-related mortality by 2030 [3,4,5]. Some limitations to improving PDAC outcomes include the lack of effective early detection strategies and a dearth of targeted therapeutic options [6,7].

Advancing the field of early detection involves understanding PDAC etiology, which can occur by studying individuals with diseases at a high risk of developing PDAC, such as those with the most common pre-malignant pancreatic cystic lesions known as intraductal papillary neoplasms (IPMNs), a sentiment in line with the National Cancer Institute’s initiative called the ‘Pre-Cancer Genome Atlas’ [6,7]. IPMNs are macrocystic lesions that can be observed by either magnetic resonance imaging (MRI) or computed tomography (CT) scans, but these lesions may take years to progress to malignant disease or may not progress at all [8,9,10]. Biomarkers for accurate diagnosis and risk stratification of IPMNs are needed so patients can be properly triaged for surveillance or surgery [11]. This will, in turn, minimize the cost of healthcare and utilization of resources for unwarranted surgical resections. Additionally, knowledge of biomarkers and pathways that can be targeted to prevent progression to PDAC from IPMNs or the most common microcystic precursor, pancreatic intraepithelial neoplasia (PanIN), is an important avenue for clinical intervention.

IPMN-derived PDAC seems to be a distinct clinical entity from ‘sporadic’ PanIN-derived PDACs (not derived from or occurring concomitantly with IPMNs), as the prognosis of IPMN-derived PDAC is often better than sporadic PDACs [12,13,14]. Furthermore, IPMN-derived PDACs may be distinct biologically. Both IPMN-derived and PanIN-derived PDAC contain similar mutation profiles, namely RAS (early) and TP53 (later) driver mutations, although a difference in the prevalence of activating KRAS mutations (for PanIN-derived/conventional) versus GNAS (for IPMN-derived) has been reported in the literature [12,13,15,16,17,18,19,20]. Although both cystic and non-cystic lesions can lead to PDAC tumors, it is unclear whether the resulting tumors have differential expression patterns. The current literature has yet to focus on differences in RNA expression between IPMN-derived and PanIN-derived lesions using a transcriptomic approach [13,15,16,17,18,21,22,23], though ITGA2 and SDC1 have been identified as potential prognostic biomarkers associated with poor survival for PDAC associated with IPMN in a recent study that did not include sporadic PDACs [18], and KLF4 was found to be mutated in >50% of low-grade foci in IPMNs with a significantly lower mutation rate in high-grade foci in another study [24]. Thus, the expression of unique molecules could be leveraged to develop imaging and/or activity probes that could be used to detect IPMN/pre-PDAC in a screening population [21]. Furthermore, differential expression analysis of IPMN- vs. PanIN-derived lesions may identify novel pathways for IPMN risk stratification via biopsy or cyst fluid.

The objective of this study was to compare, for the first time, the transcriptome of PDAC tumors arising from PanIN and IPMN-derived etiologies, with the overarching goal of informing strategies for early detection and treatment of both kinds of PDAC precursor lesions.

## 2. Results

**Figure 1:**
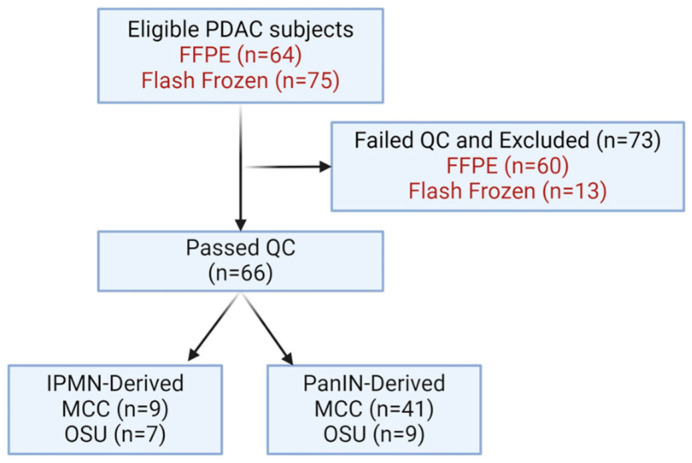
Flowchart of all eligible samples analyzed. Sample types are denoted in red font. A total of 66 samples were included in the analytic dataset (16 IPMN-derived and 50 PanIN-derived tumors). Abbreviations: FFPE, formalin-fixed paraffin-embedded; QC, quality control; MCC, Moffitt Cancer Center; and OSU, The Ohio State University.

Characteristics of the Analytic Cohort: RNAseq data were generated from tumor tissue from 139 eligible cases with a total of 66/139 samples included. Of the 73 samples that failed quality control (QC), most (*n* = 60) were from formalin-fixed paraffin-embedded (FFPE), while 13 were from flash-frozen (FF) tissues. Thus, the final analytic dataset includes 66 cases (50 from Moffitt and 16 from Ohio State University (OSU)) (Figure 1). Patients were an average of 68.3 years of age at diagnosis/time of resection (range 48–87, Table 1). Most cohort participants were males (*n* = 37, 56%), and most were non-Hispanic white (*n* = 58, 87%, Table 1). Most cases (*n* = 50/66) had conventional PanIN-derived PDAC, and 16 were IPMN-derived.

To ensure that institution (Moffitt vs. OSU) and sample-type batch effects were minimal, we performed principal component (PC) analyses to assess clustering. PC plots (PC1 vs. PC2) colored based on tumor derivation reveal no clustering according to whether the sample was IPMN or PanIN-derived (Figure S1A). No appreciable batch effects were observed according to the institution of sample origin (OSU or Moffitt, Figure S1F). PC plots based on cancer etiology (Figure S1A), sex (Figure S1B), ethnicity (Figure S1C), race (Figure S1D), or sample type (Figure S1E) also show no obvious clustering. Combined, PC1 and PC2 account for a total of 40% of the variance in the dataset.

**Figure 2:**
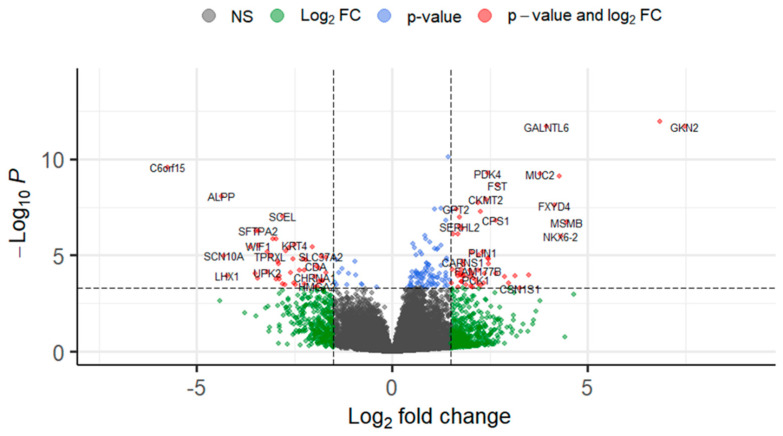
Expression profiles differ between IPMN- and PanIN-derived tumors. Figure 2 shows a volcano plot of differential expression (x-axis) for IPMN-derived versus PanIN-derived tumors. PanIN-derived tumors were used as the reference and thus have negative fold change values.

Gene expression profiles differ between IPMN-derived versus PanIN-derived PDACs: A total of 215 genes were significantly deregulated (154 upregulated and 61 downregulated, adjusted *p*-value < 0.05) when comparing expression levels of IPMN-derived tumors to our reference group, PanIN-derived PDAC tumors (Table S1). Table 2 includes an abbreviated list of these genes with a fold change (FC) of >2 or <2 and an adjusted *p*-value (Padj) < 0.01. Genes significantly upregulated in IPMN-derived tumors include gastrokinin 2 (GKN2, FC = 7.5), gastrokinin 1 (FC = 7.6), Polypeptide N-acetylgalactosaminyltransferase-like 6 (GALNTL6, FC = 4.0), mucin 2 (MUC2, FC 3.8), pyruvate dehydrogenase kinase (PDK4, FC = 2.4), and serine peptidase inhibitor Kazal type 4 (SPINK4, FC = 4.3) (Figure 2, Table 2). Alkaline phosphatase placental (ALPP, FC = −4.3) and the predicted protein-coding gene C6orf15 (FC = −5.7) were among the most significantly upregulated genes in PanIN-derived tumors.

When we analyzed KRAS and GNAS DNA mutational profiles in our cohort, we found that the proportion of KRAS mutations was significantly higher in the PanIN-derived group, and GNAS mutations were significantly more common in the IPMN-derived group (Figure S2).

**Figure 3:**
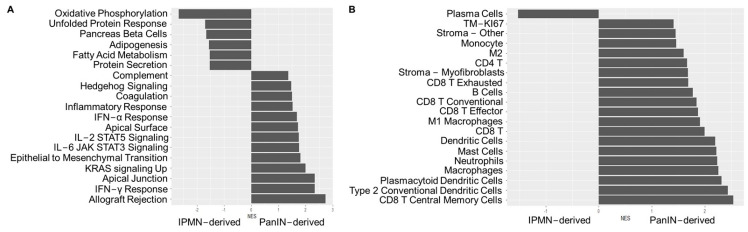
PanIN-derived tumors are enriched for genes in inflammatory pathways, whereas IPMN-derived tumors are enriched for genes in energy production/metabolism pathways. (**A**,**B**) Bar graph of the normalized enrichment score (NES) on the x-axis versus the GSEA “Hallmark” pathways (**A**) or immune cell signatures (**B**) on the y-axis. Gene sets with a *p*-value of >0.05 are excluded.

Gene set enrichment analysis (GSEA) indicates differential regulation of cellular metabolism and immune/inflammatory signaling in IPMN- vs. PanIN-derived PDAC: Normalized gene set enrichment scores (NES) from the Moffitt-OSU ORIEN/Avatar cohort were generated using either the publicly available “Hallmark” set of pathways or the immune deconvoluting gene set for PDAC (Figure 3A,B, text file S1) developed using single-cell transcriptomic data from Tumor Immune Single Cell Hub (TISCH) across 16 solid cancer types including PDAC (TIMEx) [25,26] were calculated using the GenePattern webtool and GSEA pipelines and filtered based on *p*-values (*p* < 0.05, Figure 3A,B). As shown in Figure 3A, Hallmark gene sets enriched in PanIN-derived PDACs include allograft rejection and IFN-γ response, as well as other immune/inflammatory pathways. Gene sets enriched in IPMN-derived PDACs include energy-sensing and production pathways such as oxidative phosphorylation, unfolded protein response, and adipogenesis. Immune cell type gene signatures from TIMEx were almost exclusively enriched in PDACs not derived from IPMNs and included monocytes/macrophages, neutrophils, mast cells, dendritic cells, and CD8^+^ T cells (Figure 3B). A supplementary analysis using the Reactome Webtool (version 81) [27,28,29] also demonstrates an enrichment of inflammation/immune-related genes/cell signatures in PanIN-derived PDAC, while IPMN-derived PDAC shows enrichment for metabolic/energy-related and cell cycle genes (Table S2).

The cancer genome atlas (TCGA) pancreatic adenocarcinoma (PAAD) cohort shows similarity to our findings for metabolism-linked pathways: Although tumor origin is not captured in the clinical variables for the TCGA PAAD cohort, we evaluated this publicly available dataset, which includes RNA sequencing from pancreatic tumor tissue [30,31,32] in a supervised analysis. For this clustering, we used genes found in the pathways described in Figure 3. Custom gene profiles were assigned as follows: genes in Hallmark pathways identified in our GSEA (*p* < 0.05) that were core enriched in either IPMN-derived tumors (oxidative phosphorylation, unfolded protein response, pancreas beta cells, adipogenesis, fatty acid metabolism, protein secretion) or PanIN-derived tumors (complement, hedgehog signaling, coagulation, inflammatory response, apical surface, IL-2/STAT5, IL-6/STAT3, EMT, KRAS signaling, apical junction, IFN-gamma, allograft rejection) were included if they appeared in more than one pathway (see Table S3 for a list of all included genes and the pathways in which they were core enriched). After filtering to adenocarcinoma samples, these manually curated gene sets (IPMN-derived and PanIN-derived) were used to cluster (hierarchical clustering) patient samples in the TCGA dataset.

**Figure 4:**
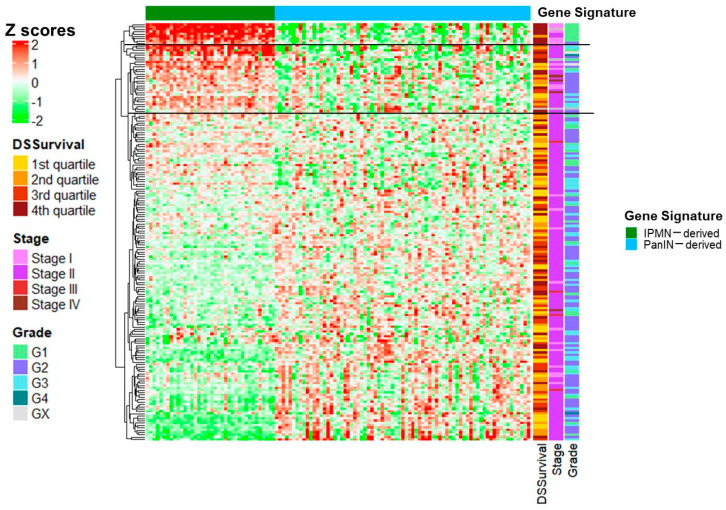
IPMN-derived metabolic gene sets cluster in The Cancer Genome Atlas data with Stage I and low-grade tumors: Heatmaps showing patient samples (y-axis) and genes in IPMN-derived and non-IPMN-derived gene sets (x-axis). Hierarchical clustering was performed on gene sets. Patients were annotated based on disease-specific survival (DSSurvival, based on quartiles), stage (AJCC), and grade (1, 2, 3, 4 or not graded (GX)).

As shown in Figure 4, we noted a small cluster of TCGA PAAD patients having upregulated tumor expression for genes in the IPMN-derived gene set, and these patients are almost exclusively stage I, low-grade (G1), and have long (based on a quartile split) disease-specific survival times. In addition, we noted a cluster of patients who demonstrate a moderate upregulation of IPMN-derived genes, and these patients show more mixed stage, grade, and survival characteristics. There was no notable PanIN-derived (immune gene) clustering based on the gene profiles from pathways identified in the ORIEN/Avatar cohort, nor was there differential enrichment of the classical/basal subtypes [33] (Figure S3A). We observed minimal clustering based on gene expression fingerprints associated with IPMN invasiveness from Huang et al. [34] or Sato et al. [35] (Figure S3B,C).

## 3. Discussion

We demonstrate that significant expression-level and pathway-level differences occur between IPMN-derived versus PanIN-derived PDAC tumors. We highlight that IPMN-derived PDAC exhibits changes in genes related to metabolism, while PanIN-derived PDAC exhibits changes in genes related to immunology. These molecular profiles have not been identified in other studies of IPMN-derived PDAC [34,35] though lipid profiles differ among cystic lesion types [36]. These findings may have implications for the early detection and treatment of these tumor types, as both metabolic dysfunction and inflammation are targetable pathways [37,38,39,40,41,42,43].

When comparing IPMN-derived versus PanIN-derived PDAC at the gene expression level, we observed significant dysregulation in many metabolism-related genes, such as PDK4 (increased) and ALPP (decreased). As expected, MUC2 was upregulated in the IPMN-derived group [44,45]. Conversely, we find that PanIN-derived PDAC was more likely to be enriched for immune/inflammatory pathways such as complement signaling, the inflammatory response, and IFN-γ signaling. Others have demonstrated similar metabolic and/or inflammatory pathway enrichment when subtyping lung [46,47], breast [37], and gastrointestinal [48,49] malignancies, though links to favorable versus unfavorable prognoses were mixed. The response to immunotherapy has so far been marginal for PDAC patients [50,51,52]. Nevertheless, our data and others [38,47,48] suggest that dysregulation of genes in energy-generating pathways may be a favorable prognostic indicator in some solid tumor types.

Interestingly, GKN2 was one of the most upregulated genes in the IPMN-derived cohort, which is in line with the literature suggesting that IPMN-derived PDAC patients have longer survival times, possibly due to earlier diagnosis [53,54]. Notably, GKN2 increases sensitivity/cell death in response to reactive oxygen species in gastric cancer cell lines [55]. Others have demonstrated that GKN2 has low expression in tumor tissues and inhibits PDAC progression [56,57]. Other genes that were found to be highly upregulated in IPMN-derived PDACs include GALNTL6, PDK4, and SPINK4. GALNTL6 catalyzes O-linked glycosylation of mucins, an enzymatic activity that is associated with cadherin switching and proliferation in PDAC [58]. PDK4 is usually not highly expressed in PDAC but is associated with cancer cachexia [59]. SPINK4 is also intriguing as it is linked to the regulation of glycolysis in colorectal cancer but is not well characterized in pancreatic cancers [60]. Regarding genes that are downregulated in IPMN-derived PDACs, decreased ALPP may be linked to others’ research indicating that the alkaline phosphatase to albumin ratio is a prognostic indicator for PDAC after curative resection [61], but not much has been published about C6orf15 except that it appears on a gene fingerprint predictive of poor survival in PDACs [62]. We note that neither KLF4 nor SDC1 were differentially expressed in our comparison [18], likely due to the fact that our comparison included PDACs derived from the PanIN pathway.

There is a complex interplay of pro- and anti-inflammatory cytokines/immune cells driving PDAC progression and drug resistance [63,64,65,66,67,68,69], and our findings demonstrate that, in comparison to IPMN-derived PDAC, PanIN-derived PDACs have a richer immune/inflammatory component. This information could be useful to stratify patients for prognosis or therapeutic regimens [70]. For example, others have shown that an increase in oxidative phosphorylation signaling promotes hypoxia-induced chemoresistance and that this could be reversed by glutaminase inhibition in an orthotopic PDAC model [71]. Yet other studies indicate that a physical activity intervention both decreases inflammatory markers and delays PDAC progression [72]. Thus, our data provide additional rationale that exercise and/or dietary interventions may be beneficial for patients with PanIN-derived PDAC [72,73,74,75].

In our GSEA, normalized enrichment scores favored the IPMN-derived PDAC for energy-sensing and energy-production pathways such as oxidative phosphorylation and lipogenesis/fatty acid metabolism), consistent with existing data [39,44,76,77,78] and with a cluster of TCGA PAAD patients who were diagnosed at an early stage and had long survival times, characteristics associated with IPMN-derived PDAC patients [12].

Regarding beneficial therapies for PanIN-derived PDACs, we find that IFN-γ-related signaling pathways are enriched in these tumors. Recently, IFN-γ has been shown to sensitize PDAC tumors to PD-1 inhibition in an animal model [79]. Thus, although the response to IFN-gamma has been mixed in other pre-clinical studies, renewed interest in these immunomodulatory therapies may be warranted [79,80]. Other groups have reported racial disparities in response to immunotherapy (with African Americans having a better response than Whites [81,82,83,84]), and racial differences in energy metabolism are well documented [85,86,87,88]. Thus, our findings also underscore the dire need for minority inclusion in future PDAC clinical trials and research efforts.

While we used a unique cohort of samples from two institutions, there are some limitations to this study. First, we were only able to obtain expression profiles for a small cohort of mostly treatment-naïve PDAC patients (*n* = 66), mainly due to issues with FFPE samples not passing QC for RNA sequencing. In addition, we were only able to confirm related cystic lesions in 16 of the cases, which is in line with published data on the incidence of IPMN-derived PDAC [8,9,10]. Furthermore, given that there were only six Hispanic or Black/African American participants (the race of two participants was unavailable), race and ethnicity could not be statistically considered in this cohort, and thus the implications for metabolic vs. inflammatory signaling enrichment by race and ethnicity are unknown. While the distribution of cases was predominantly early-stage tumors, this supports our goal to conduct investigations that would be informative for PDAC intervention.

## 4. Methods

Study population: The study population included male and female adults (≥ age 18) who consented to the Total Cancer Care (TCC) protocol [89] at Moffitt Cancer Center (Moffitt, Tampa, FL, USA) and The Ohio State University (OSU, Columbus, OH, USA) who underwent surgical resection of a pancreatic tumor between 2005 and 2020 and were pathologically confirmed to have a diagnosis of PDAC or related histology using ICD-O-3 codes 82553, 84503, 81403, 84803, 85003 and 84532. Hematoxylin and eosin (H&E)-stained slides and electronic medical records from eligible patients were analyzed by a pathologist to determine IPMN involvement. IPMNs were classified based on the Fukuoka guidelines [90]. If a pathology report noted that invasive cancer was present in association with and/or histologically contiguous with an IPMN, the PDAC was considered to be derived from the IPMN. If there was a distance from the focus of invasion and the IPMN according to the pathology report, or if no IPMN was noted, we considered a possibility of de novo origination and, therefore, were classified as PanIN-derived. Neuroendocrine tumors and metastases of pancreatic primary tumors were excluded.

Sample Handling and RNA Extraction: Surgically resected pancreas tumor tissue was retrieved from the Moffitt and OSU institutional biobanks for all eligible cases. Snap-frozen tissue aliquots were the specimen type of choice; if frozen tissue was unavailable, formalin-fixed paraffin-embedded (FFPE) tissue blocks were used. After pathological review to confirm the diagnosis, tissue specimens underwent nucleic acid extraction and sequencing at Aster Insights/M2Gen/HudsonAlpha (Huntsville, AL, USA). For RNA isolation from frozen tissue, the Qiagen RNAeasy plus mini kit was used, generating a 216 bp average insert size. For FFPE tissue, the Covaris Ultrasonication FFPE DNA/RNA kit was utilized to extract both DNA and RNA, generating a 165 bp average insert size. DV200 (fraction of RNA fragments longer than 200 base pairs) was >20, and total RNA was >20 ng for all samples except for one (5.7 ng RNA), which was analyzed prior to updated standard operating procedures (SOPs) in 2016. RNA sequencing (RNAseq) was performed using the Illumina TruSeq RNA Exome with single library hybridization, cDNA synthesis, library preparation, and sequencing (100 bp paired reads) to a coverage of 100 M total reads/50 M paired reads. Detailed SOPs for FFPE sample collection are included in Supplemental Methods.

RNAseq Analysis: Fastq files were aligned to the human genome (hs37d5) using STAR (v2.5.3a) [90]. Gene-level quantitation was performed using HTSeq (v0.6.1) [91], and QC was performed on the count’s files using standard RNAseq quality metrics (% aligned, % intronic) and visualizations.

Differential Expression Analysis: HTSeq count files were evaluated for standard metrics and visualizations such as counts per sample. Differential expression was assessed in samples using various contrasts using the “DESeq2” R package (version 3.2) and a simple 2-group model [92].

Pathway Analysis and Immune Profiling: Fragments per kilobase million (FPKM) values were subjected to gene set enrichment analysis (GSEA) using GenePattern (version B388) [93,94] and using either a publicly available universal gene set (hallmark pathways) or an immune deconvoluting gene signature for PDAC (the TIMEx tool, https://timex.moffitt.org/, accessed 2 November 2022) [25]. For further confirmation, FPKM values from the ORIEN/Avatar dataset were analyzed using the Reactome Web Tool (version 81) [27,28,29]. In this analysis, the IPMN-derived group was used as the reference group. Thus, a negative enrichment score means that these pathways were differentially enriched in this group (compared to the PanIN-derived group).

Hierarchical Clustering: As a supplemental analysis, RNA expression data from the pancreatic adenocarcinoma (PAAD) dataset from The Cancer Genome Atlas (TCGA) was downloaded from the Genome Data Commons (GDC) portal along with clinical characteristics of the participants, including survival, stage, and grade [95]. A custom gene set was generated by taking all genes in each hallmark pathway with a *p*-value of <0.05, which were core enriched (i.e., the subset of genes contributing to the leading edge of the enrichment score) in our analysis and filtering to those genes which appeared in >1 pathway to find a subset of genes in overlapping pathways. (Table S3). Hierarchical clustering was performed on scaled expression values using manually curated gene signatures and visualized using the “complexHeatmaps” R package (version 3.2) [96].

Statistical Analysis: Statistical analyses were performed using R. Chi-square tests were performed on proportion-type data. Principal Component Analysis (PCA) was performed using base R and plotted using “ggbiplot” (version 0.55) [97].

## 5. Conclusions

Enrichment in energy metabolism pathway genes was associated with IPMN-derived lesions, while enrichment in inflammation was associated with PanIN-derived lesions in our analytic cohort. Taken together, our results provide additional rationale for metabolic interventions in patients with or at risk for IPMNs [72]. Our study also suggests that inflammatory and immune pathways may be plausible targets for PanIN-associated PDAC patients but less so for those with IPMN-derived PDAC. Further studies are needed to validate these findings, better define metabolic/inflammatory signatures for non-IPMN- vs. PanIN-derived PDAC patients, and identify better therapies related to PDAC tumor origin.

## Figures and Tables

**Table 1:** Select characteristics of patients who donated pancreas tumor samples for RNA sequencing analysis.

Characteristics (*n* = 66)	
**Age at Diagnosis, Mean (Range)**	68.3 (Min 48; Max 87)
**Sex, N (%)**	
Male	37 (56.1%)
Female	29 (43.9%)
**Race &Ethnicity, N (%)**	
Non-Hispanic White	58 (87.9%)
Non-Hispanic Black	3 (4.5%)
Hispanic	3 (4.5%)
Other/missing	2 (3.0%)
**Histology, N (%)**	
Adenocarcinoma, NOS	33 (50.0%)
IPMN, non-invasive	2 (3.0%)
Invasive carcinoma of no special type	28 (42.4%)
Adenocarcinoma in situ	1 (1.5%)
Mucinous adenocarcinoma	1 (1.5%)
Adenocarcinoma with mixed subtypes	1 (1.5%)
**Grade, N %**	
*Grade 1–2*	33 (50.0%)
PanIN-derived	26 (39.4%)
IPMN-derived	7 (10.6%)
*Grade 3*	17 (25.8%)
PanIN-derived	14 (21.1%)
IPMN-derived	3 (4.5%)
**Stage, N %**	
*Early (stage I/II)*	56 (84.8%)
PanIN-derived	43 (65.2%)
IPMN-derived	13 (19.7%)
*Late (stage III/IV)*	5 (7.6%)
PanIN-derived	5 (7.6%)
IPMN-derived	0 (0%)
**Sample Treatment Status**	
Treatment Naïve	41 (31 PanIN-derived)
Neoadjuvant chemotherapy	9 (9 PanIN-derived)
**Group Description**	
IPMN-derived	16 (24.2%)
PanIN-derived	50 (75.8%)
**Survival (in months, median (LCL;UCL))**	
PanIN-derived	27 (19; 37)
IPMN-derived	36 (25; NA)

Abbreviations: LCL, lower confidence interval limit; UCL, upper confidence interval limit; min, minimum; max, maximum; and NA, not applicable. Totals may not equal 100% as some information is missing.

**Table 2:** List of dysregulated genes with an adjusted *p*-value (Padj) < 0.01 and a fold change (log2 FC) of >2 (IPMN-derived) or <2 (PanIN-derived).

Gene Name	Log2 FC	lfc SE	*p*-Value	Padj
GKN2	7.489	1.065	<0.001	<0.001
INSL4	6.838	0.961	<0.001	<0.001
C6orf15	−5.736	0.908	<0.001	<0.001
MSMB	4.464	0.857	<0.001	<0.001
ALPP	−4.376	0.759	<0.001	<0.001
NKX6-2	4.316	0.883	<0.001	0.001
SCN10A	−4.314	0.978	<0.001	0.004
SPINK4	4.280	0.695	<0.001	<0.001
FXYD4	4.161	0.747	<0.001	<0.001
GALNTL6	3.950	0.562	<0.001	<0.001
MUC2	3.775	0.610	<0.001	<0.001
ALPPL2	−3.623	0.783	<0.001	<0.001
DSG3	−3.490	0.697	<0.001	<0.001
SFTPA2	−3.431	0.683	<0.001	<0.001
WIF1	−3.417	0.733	<0.001	0.002
CLDN6	−3.187	0.708	<0.001	0.003
TPRXL	−3.117	0.708	<0.001	0.004
NCCRP1	−3.046	0.632	<0.001	0.001
TNNT1	−2.966	0.616	<0.001	0.001
PRSS33	−2.930	0.688	<0.001	0.006
PADI3	−2.901	0.693	<0.001	0.008
SCEL	−2.809	0.525	<0.001	0.000
CALB1	−2.717	0.600	<0.001	0.002
FST	2.686	0.449	<0.001	<0.001
CPS1	2.654	0.505	<0.001	<0.001
CHIT1	−2.639	0.572	<0.001	0.002
MUC21	−2.539	0.587	<0.001	0.005
KRT4	−2.493	0.532	<0.001	0.001
SCARA5	2.466	0.588	<0.001	0.007
ORM1	2.457	0.569	<0.001	0.005
PDK4	2.448	0.394	<0.001	<0.001
CA9	2.433	0.562	<0.001	<0.001
CKMT2	2.398	0.421	<0.001	<0.001
PLIN1	2.318	0.515	<0.001	0.003
KCNS1	−2.275	0.527	<0.001	0.005
PPARGC1A	2.260	0.415	<0.001	<0.001
TRPA1	2.221	0.502	<0.001	0.004
IL20RB	−2.212	0.514	<0.001	0.005
MAMDC4	2.187	0.389	<0.001	<0.001
PPP1R14D	−2.043	0.442	<0.001	0.002
LINGO4	2.027	0.452	<0.001	0.003

Abbreviations: FC, Fold change; lfcSE, log2 fold change standard error; Padj, Benjamini–Hochberg adjusted *p*-value.

## Data Availability

The data presented in this study are not publicly available due to privacy/HIPAA restrictions.

## Supplementary Materials

The following supporting information can be downloaded at https://www.mdpi.com/article/10.3390/ijms252313164/s1.
