# Supplementary material for: Molecular Pathway and Immune Profile Analysis of IPMN-Derived Versus PanIN-Derived Pancreatic Ductal Adenocarcinomas
Source: Int J Mol Sci. 2024 Dec 7;25(23):13164. doi: 10.3390/ijms252313164 (PMC11642437; doi:10.3390/ijms252313164)
Supplement: Supplementary file 1 [file ijms-25-13164-s001.zip › Supplemental Figure Legends.pdf]

Figure S1: Principal component analysis performed on sequencing data from all samples in the cohort showing principal component 1 (x-axis) versus principal component 2 (y-axis). Samples were colored by etiology (A), sex (B), ethnicity (C), race (D), sample type (E) or site (F).

Figure S2: GNAS mutations are more common in IPMN-derived PDAC and KRAS mutations are more common in PanIN-derived PDAC. Samples in the original cohort which passed QC for DNA sequencing (n=97) were analyzed for the proportion of driver mutations in either KRAS or GNAS. The left side of the figure shows a forest plot of the odds ratios for these two genes of interest. A table indicating the number of driver mutations for each gene is shown on the right.

Figure S3: Both classical and basal gene signatures are enriched in the PanIN-derived group. A) Enrichment plot for the gene signature of either the basal (left) or the classical (right) subgroups. B-C) Hierarchical clustering of our cohort based on either Huang et al. (B) or Sato et al. (C).
