# Supplementary material for: Molecular Pathway and Immune Profile Analysis of IPMN-Derived Versus PanIN-Derived Pancreatic Ductal Adenocarcinomas
Source: Int J Mol Sci. 2024 Dec 7;25(23):13164. doi: 10.3390/ijms252313164 (PMC11642437; doi:10.3390/ijms252313164)
Supplement: Supplementary file 1 [file ijms-25-13164-s001.zip › Supplemental Methods.pdf]

Supplemental Methods:

Whole exome sequencing (WES): WES was performed by Aster Insights using samples paired to the RNAseq analysis (See main manuscript Table 1) from the Oncology Research Information Exchange Network (ORIEN). All DNA sequences that passed QC were used. DNA was extracted from patient tumors and libraries were prepared using hybrid capture and an enhanced Integrated DNA Technology WES kit (38.7 megabases). Library hybridization was performed at either single or 8-plex and sequenced on an Illumina (San Diego, CA) NovaSeq 6000 instrument generating 100 base pair paired reads. [1]

FASTQ files were analyzed and variant allele frequency was calculated using the methods described in [1].

1. Fenstermacher, D.A., et al., *Implementing personalized medicine in a cancer center.* Cancer J, 2011. **17**(6): p. 528-36.
