# Supplementary figures and images for: Molecular Pathway and Immune Profile Analysis of IPMN-Derived Versus PanIN-Derived Pancreatic Ductal Adenocarcinomas
Source: Int J Mol Sci. 2024 Dec 7;25(23):13164. doi: 10.3390/ijms252313164 (PMC11642437; doi:10.3390/ijms252313164)

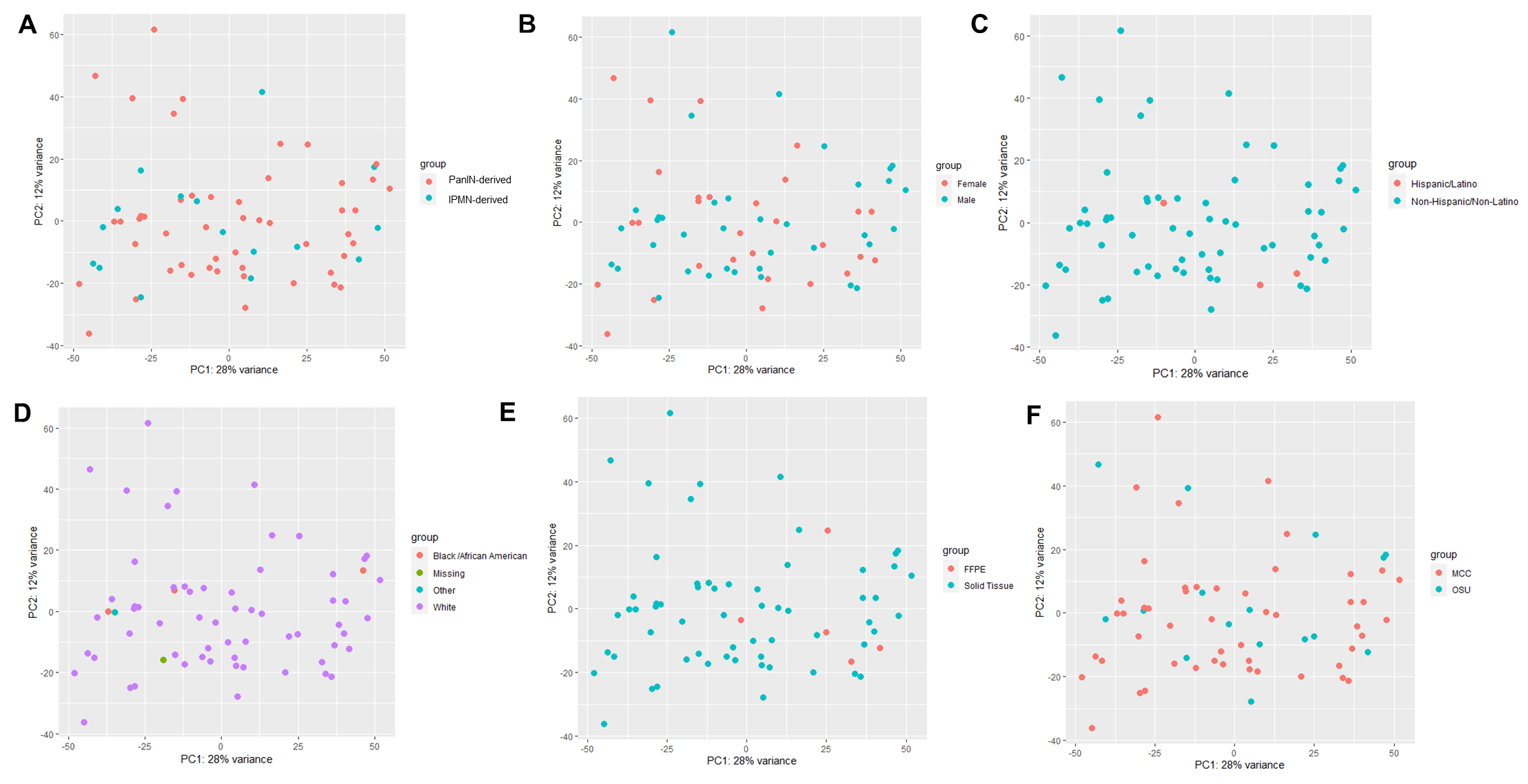

Supplement: Supplementary file 1 [file ijms-25-13164-s001.zip › FigureS1.tif]

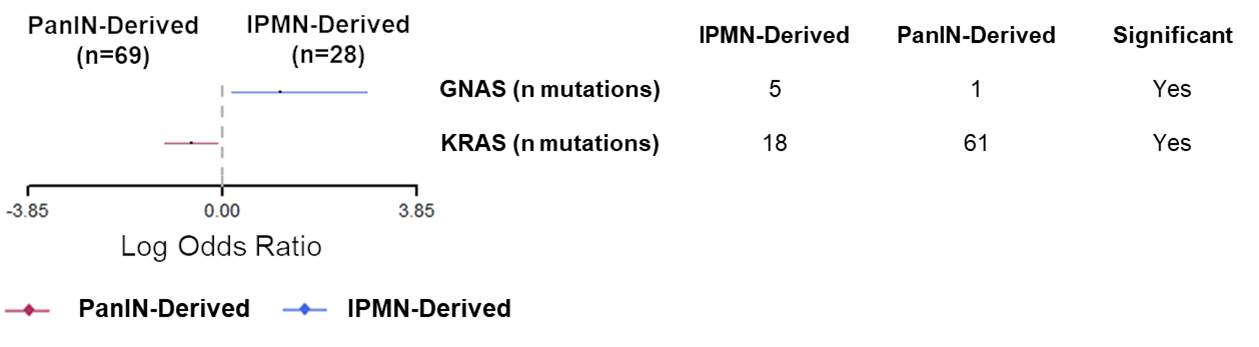

Supplement: Supplementary file 1 [file ijms-25-13164-s001.zip › FigureS2.tif]

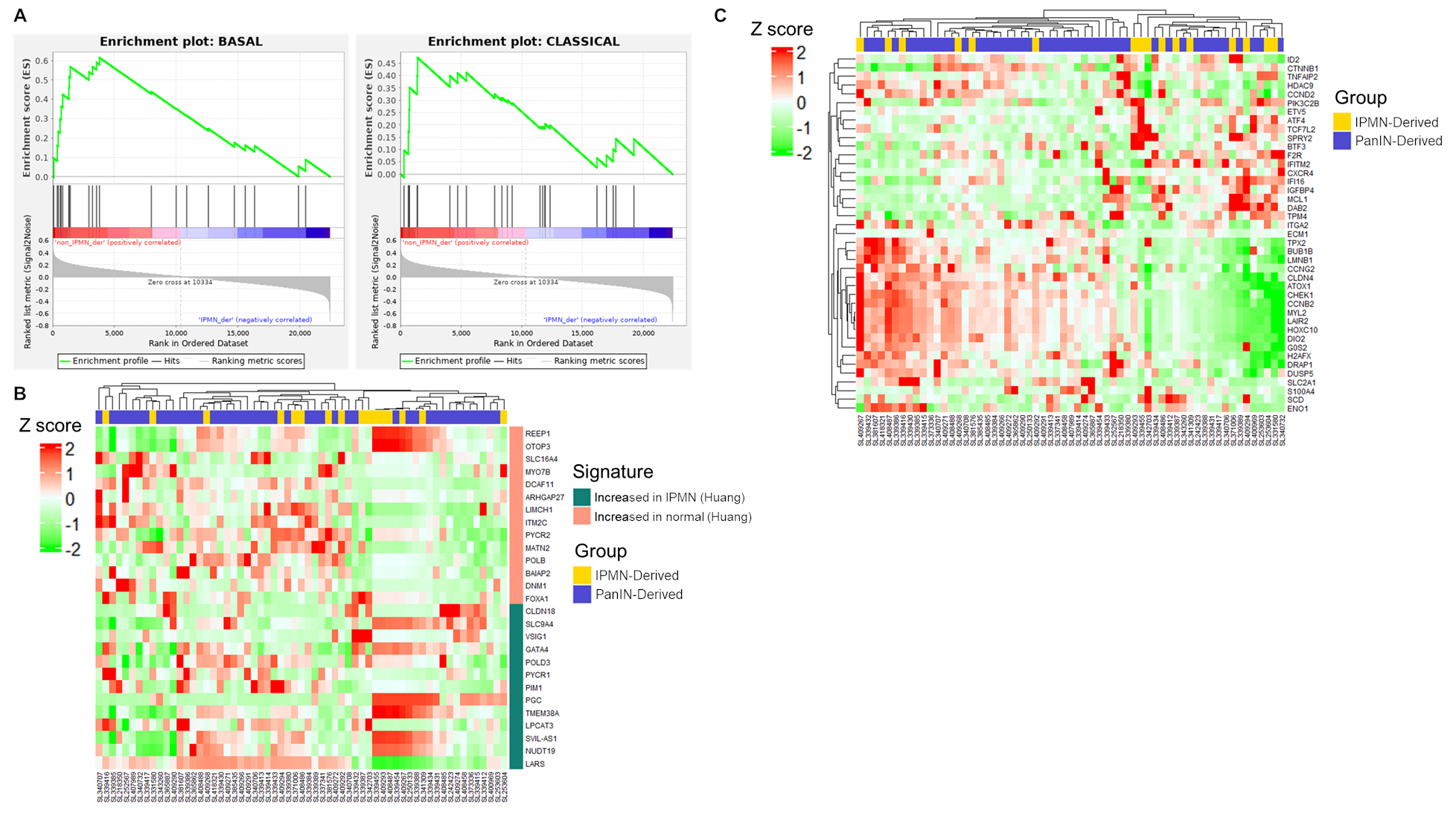

Supplement: Supplementary file 1 [file ijms-25-13164-s001.zip › FigureS3.tif]
